# Silymarin attenuates mycophenolate mofetil-induced duodenal disorders in rats

**Published:** 2014

**Authors:** Hassan Malekinejad, Sanaz Sheikhzadeh, Rahim Hobbenaghi

**Affiliations:** 1 ^*‎*^*Department of Pharmacology & Toxicology, Faculty of Veterinary Medicine, Urmia University, Urmia, I. R. Iran *; 2 *Department of Pathology, Faculty of Veterinary Medicine, Urmia University, Urmia, Iran, I. R. Iran *

**Keywords:** *Antioxidant*, *Combination drug therapy Gastrointestinal*, *Protective effects*, *Silymarin*

## Abstract

**Objective**: The protective effect of silymarin (SMN) on mycophenolate mofetil (MMF)–induced duodenal disorders was investigated.

**Materials and Methods: **Forty-two Wistar rats were assigned to seven groups including control and test groups. The control animals received saline and the test animals were treated with MMF (30 mg/kg, orally) and saline, MMF and SMN (25, 50, and 100 mg/kg, orally), MMF and Celecoxib (CLX, 50 mg/kg, orally), and MMF and SMN plus CLX for 14 consecutive days. The antioxidant status and myeloperoxidase activity were determined and the histopathological examinations on duodenal section also were performed.

**Results:** Biochemical analyses revealed that SMN and CLX individually and in combination therapy could reduce the MMF-increased nitric oxide (NO) content, myeloperoxidase (MPA) activity, and malondialdehyde (MDA) level, while the MMF-reduced level of total thiol molecules (TTM) was increased significantly (p<0.05) by given compounds. Concurrent administration of SMN and CLX resulted in a synergistic effect on the reduction of MDA level and MPO activity. SMN and CLX were able to improve the MMF-induced histopathological damages including the villus atrophy and inflammatory cells infiltration.

**Conclusion**: Our data suggest that the MMF-induced duodenal disorders may attribute to the elevated NO and MDA levels and myeloperoxidase activity that resulted in pathological injuries. Moreover, the biochemical alterations and histopathological injuries due to MMF administration were reduced by SMN alone or in combination with CLX indicating its protective effect.

## Introduction

Mycophenolate mofetil (MMF) as an immunosuppressive pro-drug has been used to prevent graft rejection over the last decade. MMF is rapidly metabolized into its active metabolite - mycophenolic acid (MPA) - which inhibits non-competitively the rate-limiting enzyme of inosine 5-monophosphate dehydrogenase (Allison and Eugui, 2000 ▶). It has been reported that in contrary to other immunosuppressors such as azathioprine, cyclosporin A, and tacrolimus, MPA kills lymphocytes via activating the caspase-independent pathway (Moreau et al., 2008 ▶). Despite the progress made by using anti-rejection agents including MMF, physicians reported the short- and long-term adverse effects related to these drugs. Furthermore, one of the common adverse effects, which have been reported in organ transplanted patients, is the gastrointestinal (GI) disorders (Ponticelli and Passerini, 2005 ▶). 

The MMF-induced GI disorders could be seen in the entire gastrointestinal tract and in various severities. GI-associated clinical symptoms such as diarrhea, nausea, abdominal pain, vomiting, GI bleeding, dysphagia, anemia, and hematemesis have been reported between 1 month to 10 years post-transplantation. Clinical manifestations are accompanied by pathological findings including chronic peptic duodenitis, active duodenitis, celiac-like features in the duodenum, chemical gastropathy, erosion in the stomach, reactive epithelial changes, active esophagitis, ulceration, and erosion in the esophagus (Nguyen et al., 2009 ▶). Despite the fact that acute colitis is rare due to MMF administration, there are some reports indicating severe colitis in children with histological features similar to inflammatory bowel disease and graft versus host disease (Phatak et al., 2009 ▶). 

Previously, we showed that the MMF-induced GI disorders are largely related to local inflammatory reactions, which were highlighted with increased nitric oxide (NO) and myeloperoxidase activity in the gastrointestinal tract (Malekinejad et al., 2011 ▶). Following high rate of survival in organ received patient due to MMF administration, the further step would be an attempt to minimize the MMF-induced side effects including GI disorders. In this study, we aimed to investigate the protective effect of silymarin (SMN) as a known antioxidant with anti-inflammatory effect on the MMF-induced GI disorders **(**El-Lakkany et al., 2012 ▶**; **Ashkavand et al., 2012 ▶**). **

SMN as the main constituent of the extract from seeds of the milk thistle (*Silybum marianum*), is used globally as a hepatoprotective remedy in human medicine (Flora et al., 1998 ▶). Moreover, the protective effect of celecoxib as a selective cyclooxygenase-2 inhibitor and anti-inflammatory agent alone and in combination with SMN also was studied. 

## Materials and Methods

**Chemicals**

Silymarin (SMN, S 0292), 5.5’-dithiobis-2-nitrobenzoic acid (DTNB), N-(1-naphtyl) ethylendiamine.2HCl (NED), hexadecyl trimethyl ammonium bromide, and tetramethyl benzidine were purchased from Sigma Chemical Co. (St Luis, MO, USA). N-(1-naphthyl) ethylenediamine.2HCl was obtained from Sigma-Aldrich (Germany). Thiobarbituric acid, phosphoric acid (85%), dimethyl sulfoxide (DMSO), sodium nitrite, and ethanol were purchased from Merck (Germany). N-Butanol was obtained from Carl Roth, GmbH Co. (Germany). Phosphoric acid, dimethyl sulfoxide (DMSO) and ethanol were obtained from Merck (Germany). Sulfanilamide was purchased from ACROS, New Jersey (USA). All other chemicals were commercial products of analytical grade. Commercially available standard kit was used for determination of alkaline phosphatase (ALP, 744, Man Inc. Tehran, Iran).

MMF was provided by Hoffman La Roche, (Basel, Switzerland) and was suspended in saline containing 0.4% Tween 80, 0.9% benzylalcohol (Watanabe et al., 2006 ▶). SMN was dissolved primarily in small volume of ethanol and then adjusted to the final volume using saline to reach the ethanol percentage below 5% of final volume. Celecoxib (CLX, Darou Pakhsh Pharmaceutical Co., Tehran, **Iran*)*** was obtained from a local drug store (Urmia, Iran) and was suspended in saline. 

**Animals and experimental design**

Forty-two adult female Wistar rats (200-220 g) were obtained from the animal resource of the Faculty of Veterinary Medicine, Urmia University. The rats were acclimatized for one week and had free access to food and water during adaptation and experimental periods. The experimental protocols were approved by the ethical committee of Urmia University in accordance with principles of laboratory animal care (NIH publication no. 85-23, revised 1985). Animals were assigned into control and test groups (n=6). 

Animals in the test group were subdivided to following groups:

- MMF group: animals in this group received MMF (30 mg/kg, b.w., orally, every day at 15:00 PM).
- SMN 25 group: animals in this group received MMF (30 mg/kg, b.w., orally) and SMN (25 mg/kg/day, orally and every day at 9:00 AM).
- SMN 50 group: animals in this group received MMF (30 mg/kg, b.w., orally) and SMN (50 mg/kg/day, at 9:00 AM).
- SMN 100 group: animals in this group received MMF (30 mg/kg, b.w., orally) and SMN (100 mg/kg/day, at 9:00 AM).
- CLX group: rats in this group received MMF (30 mg/kg, b.w.) and (50 mg/kg CLX, orally every day at 09:00 AM).
- SMN+CLX group: animals in this group received MMF (30 mg/kg, b.w., orally and at 15:00 PM), SMN (50 mg/kg/day, orally and at 09:00 AM) and CLX (50 mg/kg, orally at 10:00 AM).

The control group received only saline (0.9%, 5 ml/kg) containing the same amount of the test compound solvent during the 14-day experiment period. Animals received saline and/or test compounds via gastric gavage. The selected dose levels for MMF and SMN were based on our previous reports (Malekinejad et al., 2011 ▶: Malekinejad et al., 2012 ▶). 

**Serum preparation and tissue samples collection**

On day 15 following a light anesthesia, which was induced by diethyl ether, blood samples were collected directly from the heart. After one hour at room temperature, the samples were centrifuged at 3000 × g for 10 min to obtain the serum. The serum samples then stored at -20 °C for further analyses. 

The anesthetized animals were euthanized using CO_2_ gas in a special device and immediately the macroscopically abnormal looking (congested and gaseous) sections of the duodenum were removed and rinsed with chilled saline. The samples were then divided into two parts which the first part was fixed in 10% formalin in phosphate buffer saline for pathological examinations and the second part was snap frozen in liquid nitrogen and kept in -70 °C till further biochemical analyses. 

**Measurement of serum level of alkaline phosphatase (ALP)**

Serum level of ALP was measured using commercially available standard kit (744, Man Inc. Tehran, Iran) and according to manufacturer's instructions. 

**NO measurement**

Total nitrate/nitrite content of duodenal tissue was measured according to the Griess reaction (Green et al., 1982 ▶). In Griess reaction, nitric oxide is rapidly converted into more stable nitrite, which in an acidic environment nitrite is converted to HNO_2_. In reaction with sulphanilamide, HNO_2_ forms a diazonium salt, which reacts with N-(1-naphthyl) ethylenediamine. 2HCL to form an azo dye that can be detected by absorbing at 540 nm wavelength. The NO content of the examined organs was expressed as nmol per mg of protein in samples. 

**Malondialdehyde (MDA) determination**

To determine the lipid peroxidation rate in the control and test groups, MDA content of the duodenal samples was measured using the thiobarbituric acid (TBA) reaction as described previously (Niehaus and Samuelsson, 1968 ▶). Briefly, 0.2-0.3 g of the samples were homogenized in ice-cooled KCl (150 mM) and then the mixture was centrifuged at 3000 ×* g* for 10 min; 0.5 ml of the supernatant was mixed with 3 ml phosphoric acid (1% V/V) and then after vortex mixing, 1 ml of 6.7 g L^−1^ TBA was added to the samples. The samples were heated at 100 °C for 45 min, and then chilled in ice. After addition of 3 ml N-butanol, the samples were centrifuged at 3000 ×* g* for 10 min. The absorbance of supernatant was measured spectrophotometerically (Pharmacia, Novaspec II, Biochrom, England), at 532 nm and the amount of lipid peroxidation end product was calculated according to the simultaneously prepared calibration curve using MDA standards. The amount of MDA was expressed as nmol per mg of protein. The protein content of the samples was assessed based on Lowery method (Lowry et al., 1951).

**Measurement of total thiol molecules (TTM)**

Total sulfhydryl level in the duodenal section was measured as described previously (Ranjbar et al., 2006 ▶). Briefly, 0.3-0.4 g of the duodenal samples were homogenized in ice-cold KCL (150 mM), and the mixture was centrifuged at 3000 ×* g* for 10 min. To 0.2 ml of the supernatant of tissue homogenate, 0.6 ml Tris-EDTA buffer (Tris base 0.25 M, ethylendiamintetraacetic acid 20 mM, pH 8.2) and thereafter 40l 5.5’-dithiobis-2-nitrobenzoic acid (10 mM in pure methanol) were added. The final volume of this mixture was made up to 4.0 ml by an extra addition of pure methanol. After 15 min incubation at room temperature, the samples were centrifuged at 3000 × g for 10 min and ultimately the absorbance of the supernatant was measured at 412 nm. The TTM capacity was expressed as nmol per mg of protein in samples. The protein content of the samples was measured according to the Lowry method (Lowry et al., 1951).

**Myeloperoxidase activity**

Myeloperoxidase (MPO) activity, as a biochemical indicator of neutrophil infiltration into the gastrointestinal tissue, was measured in collected duodenal region of the GUT samples as previously described (Cuzzocrea et al., 2003 ▶). In brief, the specimens were homogenized in 10 mM potassium phosphate buffer (pH 7.0) containing 0.5% hexadecyl trimethyl ammonium bromide and centrifuged at 20,000 × g for 30 min at 4 ºC. A solution of 1.6 mM tetramethyl benzidine and 0.1 mM H_2_O_2_ was added and reacted with an aliquot of the supernatant. Rate of change in optical density was measured at 650 nm spectrophotometrically. One unit of MPO activity was defined as degrading 1 μmol of H_2_O_2_ per min at 37 ºC and was expressed as units per milligram of tissue sample (U/mg tissue).

**Histopathological investigations**

The tissue samples, which previously had been preserved in 10% buffered formaldehyde, were embedded in paraffin and 5-6 µm sections were cut using a rotary microtome and stained with hematoxylin and eosin for investigation under light microscope. To evaluate the level of damages following exposure to MMF and the protective effects of test compounds, indices including leukocyte infiltration, shortened villi, and villous atrophy were scored numerically. The villi height to crypt depth ratio was determined using special computerized program of image analyzing system (Olympus, Shinjuku Monolith, Version 1.7, Tokyo, Japan). For each animal in the test and control groups, at least three slides from the duodenum were prepared and the averages of scored marks were analyzed. To count the mono- and poly-morphonuclear cells, 45 microscopic fields were randomly chosen in the lamina properia part of duodenal region and at the 400 × magnification the cells were counted. The histopathological studies were conducted by a pathologist who was unaware of the study purposes. 

**Statistical analyses**

The mean and standard deviation of the measured parameters were calculated. The results were analyzed using Graph Pad Prism software (version 2.01. Graph Pad Software Inc. San Diego, California, USA). The comparisons between groups were made by analysis of variance (ANOVA) followed by Bonferroni post-hoc test. p<0.05 was considered statistically different.

## Results

**SMN and CLX recovered the MMF-reduced body weight gain and lowered the MMF-elevated ALP level**

**Figure 1:**
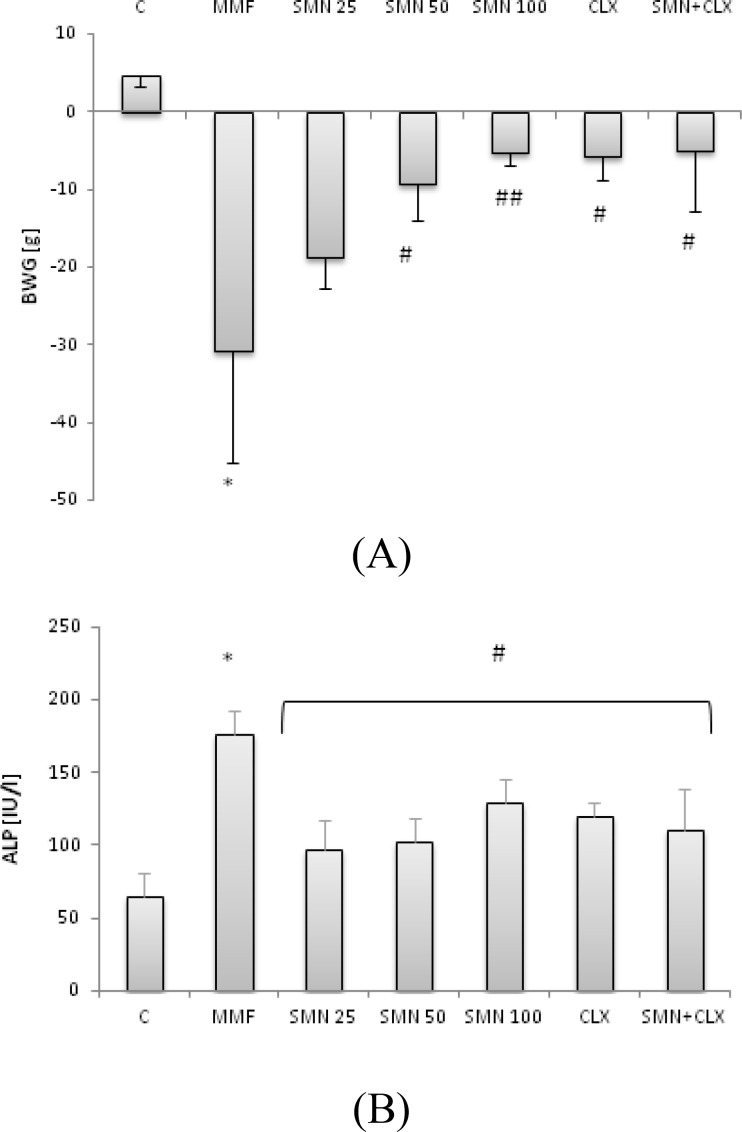
Effect of SMN and CLX on: (A) MMF-reduced body weight gain (g) and (B) MMF-increased serum level of ALP, SMN at all three given concentrations recovered significantly (p<0.05) the MMF-reduced body weight gain (BWG) and lowered the serum level of ALP. No synergistic effect was obtained between SMN and CLX. Data is given as mean±SD (n=6). Star indicates a significant difference between MMF-received group and the control group, # (p<0.05), and ## (p<0.01) represent significant differences between the MMF-treated group and other treated groups

The body weight gain (BWG) in the animals that received MMF for 14 days reduced significantly (p<0.01) in comparison with the control group, while SMN- and CLX-treated groups showed remarkable improvement in the MMF-reduced BWG. Co-administration of CLX and SMN improved the MMF-reduced BWG, nevertheless no synergism between two compounds on BWG could be achieved (Figure 1-A). SMN at given concentrations in a dose-independent fashion and CLX could lower the serum level of ALP significantly (p<0.05). No synergistic effect between SMN and CLX was obtained (Figure 1-B). 

**SMN reduced the MMF-enhanced Myeloperoxidase activity and NO level**

**Figure 2:**
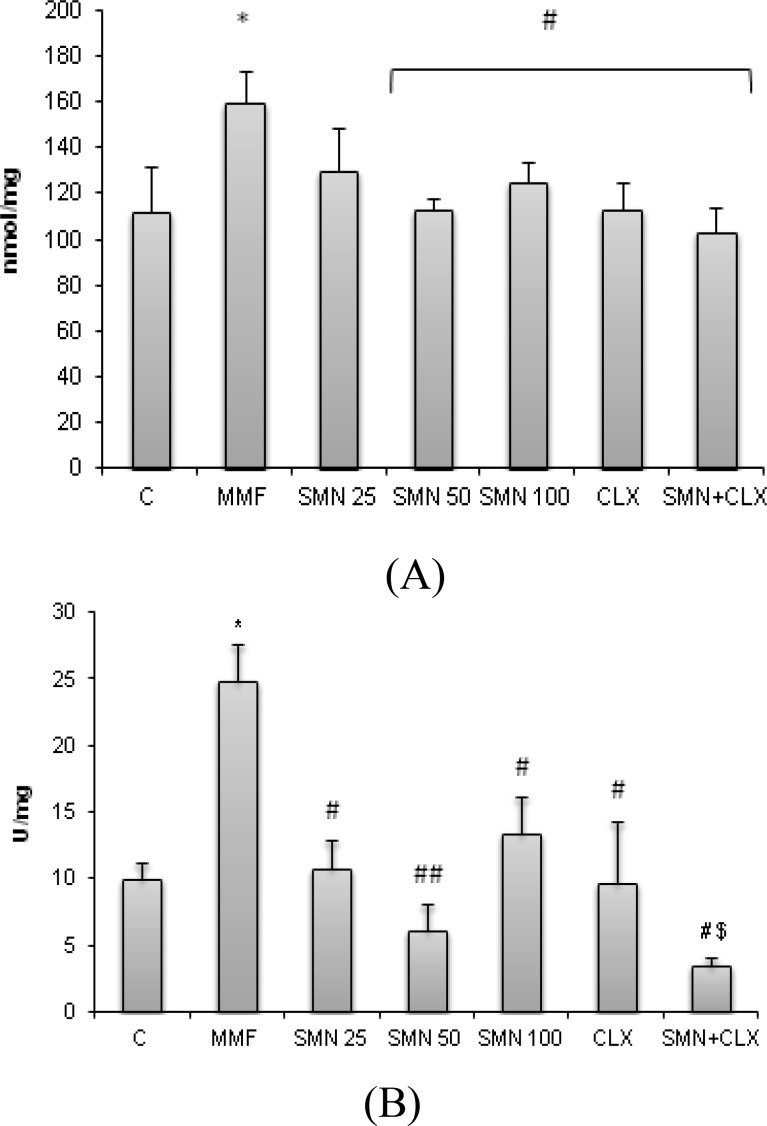
Effect of SMN and CLX on MMF-induced (A) NO level and (B) MPO activity in the duodenal region. SMN and CLX reduced the NO level and no synergistic effect was obtained between two test compounds. Both SMN and CLX reduced the MPO activity in the duodenal and the maximum reduction was observed when two compounds were administered simultaneously. Data are given as mean±SD (n=6). Star indicates a significant difference between MMF-received group and the control group, # (p<0.05), ## (p<0.01) represent significant differences between the MMF-treated group and other treated groups, and #$ shows a statistically synergistic effect between two compounds

The NO content of duodenal region in the MMF-received animals was elevated. On the contrary, SMN and CLX reduced the NO level in the duodenal tissue, with maximum reduction at 50 mg/kg dose level of SMN. Although maximum reduction in the NO-content of duodenal tissue was obtained in animals which received both test compounds simultaneously, no synergistic effect was obtained between SMN and CLX statistically (Figure 2-A). The MMF administration for 14 days resulted in a significant elevation of myeloperoxidase activity in the duodenal tissue (p<0.05). SMN and CLX individually and in combination therapy lowered the MPO activity as a biomarker of neutrophil infiltration. SMN individually at 50 mg/kg dose level exerted the strongest effect on the reduction of MPO activity. Moreover, SMN and CLX showed a synergestic effect on the reduction of MPO activity (Figure 2-B). 

**SMN attenuated the MMF-elevated MDA content and protected from the MMF-induced TTM depletion**

To analyze the antioxidant status following 14-day MMF administration and providing various treatment approaches for protection from the MMF-induced duodenal disorders, the rate of lipid peroxidation and total thiol molecules (TTM) of duodenal region were measured. Results showed that the MDA content of duodenal region in the MMF-received group was significantly (p<0.05) elevated, while SMN and CLX both lowered the lipid peroxidation rate. SMN was able to reduce the MDA level remarkably at 50 mg/kg dose level when it was given individually. 

**Figure 3:**
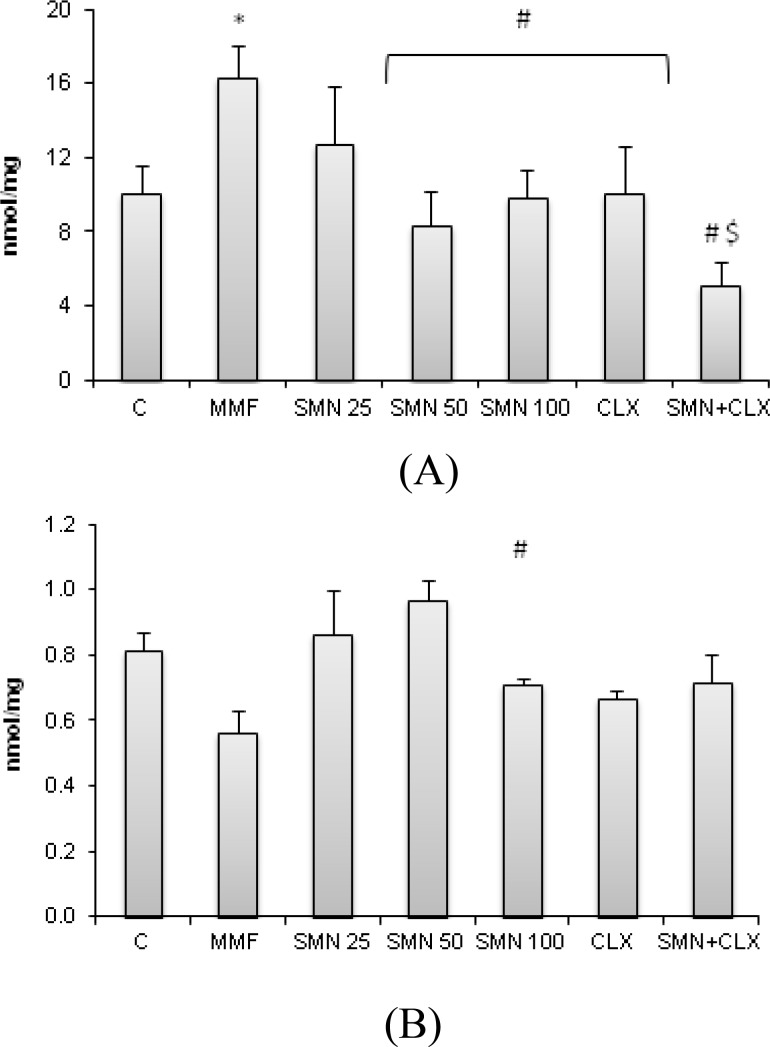
Effect of SMN and CLX on MMF-induced (A) MDA level and (B) TTM content in the duodenal region. Both SMN and CLX reduced the MDA level in the duodenal and the maximum reduction was observed when two compounds were administered concurrently. SMN and CLX reduced the TTM depletion however no synergistic effect was obtained between two test compounds. Data are given as mean±SD (n=6). Star indicates a significant difference between MMF-received group and the control group, # represents significant differences between the MMF-treated group and other treated groups, and #$ shows a statistically synergistic effect between two compounds

Co-administration of CLX and SMN showed a synergistic effect on the MDA reduction (Figure 3-A). TTM concentration was significantly (p<0.05) reduced in the MMF-received animals, while SMN at 50 mg/kg dose level prevented from thiol molecules depletion. CLX administration resulted in a slight but significant prevention from the MMF-induced TTM depletion. We failed to show any synergism between SMN and CLX in the prevention of TTM depletion (Figure3-B). 

**SMN prevented from the MMF-induced villus atrophy and attenuated the mono- and poly-morphonuclear inflammatory cells infiltration in the duodenal region**

**Figure 4:**
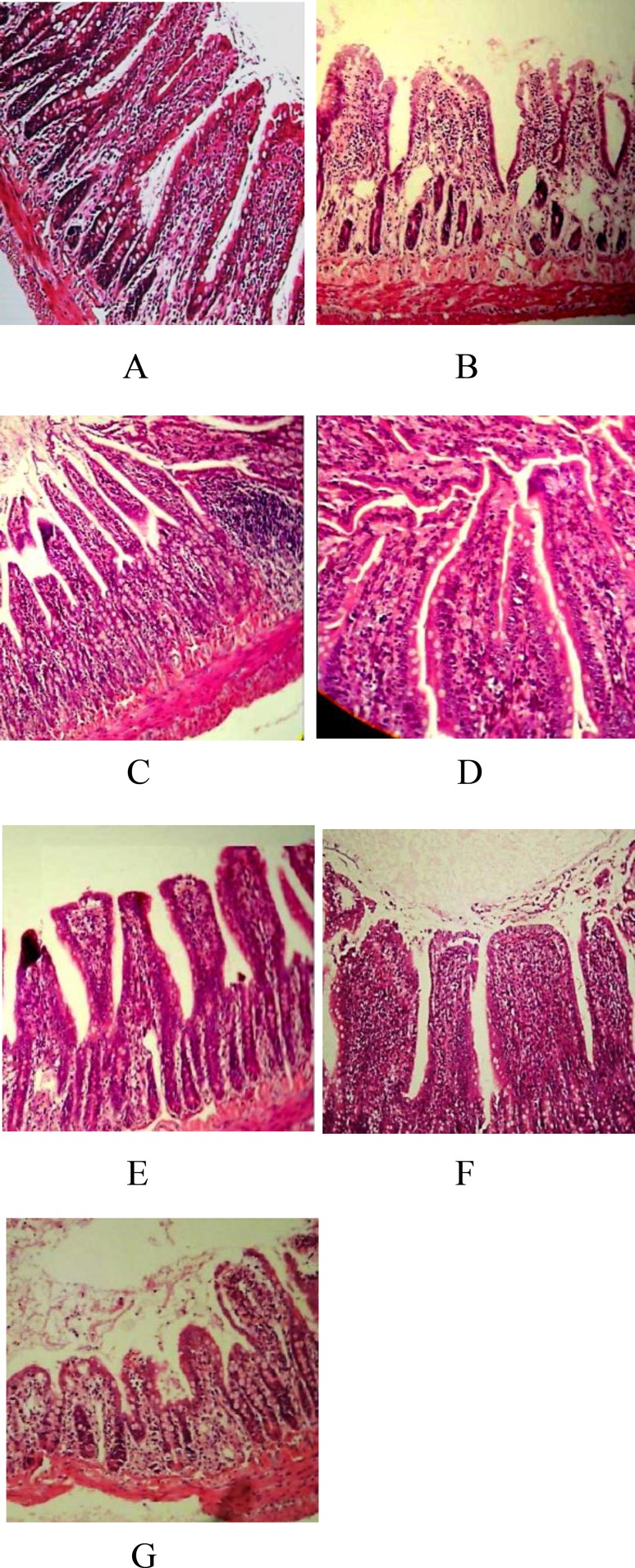
Photomicrograph of the rat’s duodenal region in the animals that received (A) saline normal and served as control group, (B) MMF at 30 mg/kg dose level, (C, D, and E) MMF and SMN at 25, 50, and 100 mg/kg dose level, respectively, (F) MMF and CLX, and (G) MMF and SMN plus CLX. With hematoxylin and eosin staining and original 100 × magnification, the villus atrophy and infiltration of inflammatory cells in lamina properia in the MMF-received group and attenuation of both pathological characters in SMN- and CLX-treated groups are observed

**Figure 5:**
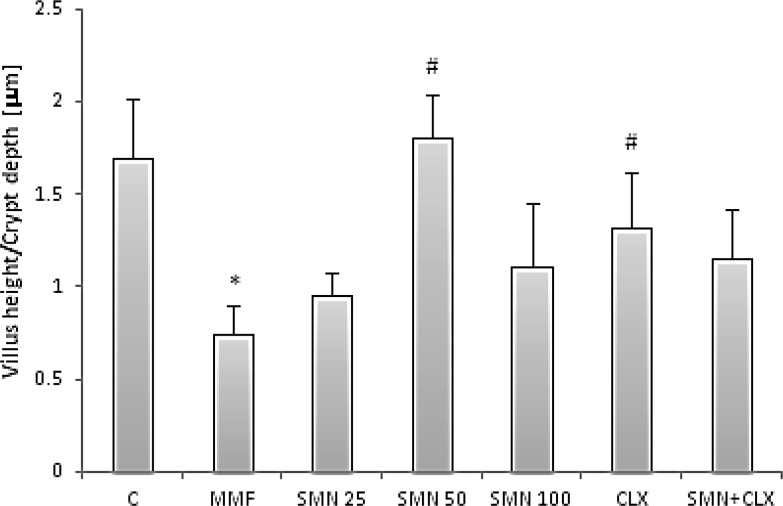
Effect of SMN and CLX on MMF-induced changes in the villus height to crypt’s depth ratio. Data are given as mean±SD (n=6). Star indicates a significant difference between MMF-received group and the control group and # represents significant differences between the MMF-treated group and other treated groups

The histopathological examinations of the duodenal samples in the control group showed no remarkable pathological changes (Figure 4-A). The irregular surface of epithelium, villus atrophy, crypt disarray, and mono- and poly-morphonuclear inflammatory cell infiltration in the lamina propria were observed in the MMF-received animals (Figure 4-B). The villi height to crypt depth ratio was determined and the results indicated that the MMF administration resulted in a significant reduction (p<0.05) of the ratio (Figure 5). SMN at 50 mg/kg dose level and CLX protected from the MMF-induced villus atrophy. In addition, administration of SMN at 25 and 50 mg/kg dose levels, CLX alone and in combination with SMN resulted in a remarkable reduction of the MMF-induced inflammatory cells infiltration (Figure 4-C, D, E, F, and G). The type and density of inflammatory mono- and poly-morphonuclear cells are depicted in Table 1.

**Table 1:** Effect of SMN and CLX on the MMF-induced inflammatory cell infiltration in the lamina propria region of duodenum

**Groups**	**Lymphocytes**	**Neutrophiles**	**Plasma Cells**
**C**	19.0 ± 6.4	1.4 ± 0.5	4.7 ± 1.5
**MMF**	71.4 ± 4.3*	3.0 ± 0.8*	18.7 ± 5.1*
**SMN 25**	31.3 ± 5.1#	2.4 ± 0.5	8.0 ± 1.8#
**SMN 50 **	34.0 ± 1.7#	2.0 ± 0.7	7.0 ± 1.4#
**SMN 100 **	49.7 ± 7.2#	2.8 ± 0.8	15.3 ± 2.3
**CLX**	32.4 ± 3.1#	1.4 ± 0.5#	9.3 ± 2.2#
**SMN + CLX**	23.0 ± 5.2# $	1.5 ± 0.6 #	9.0 ± 1.7#

Values represent mean±SD and n=6 in each group. Stars show a significant difference between the control and MMF-received animals,

# represents significant differences between the MMF-treated group and other treated groups, and

#$ shows a statistically synergistic effect between two compounds.

## Discussion

The current study showed that the MMF-induced duodenal disorders are accompanied with severe inflammatory reactions characterized by mono- and poly- nuclear inflammatory cells infiltration in the lamina propria of the duodenum. Moreover, the high level of produced MDA and remarkable elevation of MPO activity confirmed the lipid peroxidation and neutrophil infiltration as two inflammatory biomarkers. NO content increase and TTM depletion in the duodenal region of the MMF-received animals also indicated that both nitrosative and oxidative stress are involved in the MMF-induced duodenal disorders. Additionally, this study showed that SMN and CLX in individual and combination therapies are able to protect from the MMF-induced GI damages. 

In some biochemical and histopathological examinations, a synergistic effect between SMN and CLX was obtained. Undoubtedly, GI disorders which are clinically characterized by reversible abdominal pain and diarrhea, are the dominant side effects of the MMF administration as an immunosuppressive agent (Gozdowska and Urbanowicz, 2009 ▶). Hitherto, the mechanism of GI damages has not been fully clarified. In our previous study, we demonstrated an elevation of NO level and acute phase proteins in the MMF-treated rats (Malekinejad et al., 2011 ▶). In the current study, in addition to clarifying other mechanisms in the MMF-induced duodenal toxicity, the protective effect of SMN as a natural antioxidant and CLX as well-tolerated NSAIDs was examined. 

We found that the MMF-received animals showed a significant body weight (BW) loss compared with the control animals, which may be related to anorexia, low feed intake, and more importantly to the MMF-induced GI damages. Those animals which concurrently with MMF received SMN and/or CLX individually or in combination, showed a remarkable improvement in body weight gain, suggest that these compounds either increased the feed intake or lowered the MMF-related GI damages and consequently improved the nutrient absorption through the GI tract.

The effect of MMF via CNS on the feed intake has not been reported, yet. Therefore, the observed BW loss due to the MMF-administration may be attributed to the local injuries, which likely reduced the intestinal nutrient absorption. Similar clinical symptoms including body weight loss have been reported in human patients who after organ transplantation received MMF for a long time (Herrero et al., 2007 ▶). 

The significant elevation of ALP in the MMF-received animals indicates an inflammation, which indirectly also affects the feed intake in involved animals. Previous studies in rodents have demonstrated that the ALP elevation could be a biomarker of intestinal inflammation. Moreover, the increase of ALP activity in enterocytes has been microscopically demonstrated in the dextran sulfate sodium-induced model of inflammation (Renes et al., 2002 ▶; Sánchez de Medina et al., 2004 ▶). 

In this study, two histopathological points of villus height to crypt depth ratio and mono- and poly-nuclear inflammatory cells infiltration in the MMF-received animals and concurrently in the protective compounds-received rats were highlighted. Our computer-assisted program to measure the villus height and crypt depth ratio showed that MMF significantly declined the ratio. This finding was strongly supported by light microscopic examinations, which demonstrated remarkable villus atrophy in the MMF-received animals suggesting a crucial role of villus atrophy in the MMF-induced GI disorders. Another supportive finding of the current study is that in the MMF-received animals, recruitment of leukocytes including lymphocytes, neutrophils, and plasma cells in the duodenal region were significantly increased. The later issue may reflect an inflammatory reaction in the region. 

The villus atrophy as a long recognized histopathological manifestation of the MMF-induced GI disorders has been reported. The reason for villus atrophy may be related to the enterocytes dependency on the *de novo *pathway of purine synthesis (Behrend, 2001 ▶). Our findings in this study highlighted the cellular events which are involved in the MMF-induced inflammatory reactions in upper part of GI. The MMF-induced inflammatory reactions in colitis form have been reported in human patients with histopathological features of lamina propria edema, increased lamina propria inflammation, and damaged and dilated crypts (Parfitt et al., 2008 ▶). The type of present cells in the lamina propria may indicate the chronicity of events which are taking place in the inflamed organ. For example, the dominancy of lymphocytes and plasma cells suggest a sub-chronic to chronic situation after 14-day MMF administration. 

Next to the histopathological examinations, biochemical analyses such as assessment of oxidative/nitrosative stress biomarkers which were determined by measuring the TTM and NO levels and also the produced MDA level in the duodenal region support the MMF-induced damages. The elevated level of NO and MDA and simultaneously reduced level of TTM indicate locally occurred oxidative/nitrosative stress in the MMF-received animals. It seems that MMF by reducing the TTM level as an important source of antioxidant capacity and at the same time by enhancing the NO content of the duodenal region facilitates events which end up with remarkable lipid peroxidation. In the current study, to clarify the pathogenesis of MMF-induced damages, the MPO activity was measured as a biomarker of neutrophil infiltration. Indeed, our results showed a significant elevation of MPO activity in the MMF-treated animals. The increased MPO activity may be accounted as a strong source of elevated NO content in the damaged tissues. 

The contribution of neutrophils in the production of NO in the initial phase of murine asthma and in the polyethylene oxide-modified polyurethane-induced neutrophil activation which resulted in a great NO production, have been previously reported (Fernandes et al., 2007 ▶; Patel et al., 2007 ▶). NO itself and in combination with superoxide anion may promote the MMF-induced damages in the duodenal region. There is evidence indicating that iNOS as one of the key enzymes in the NO production is up-regulated in the patients with Alzheimer’s disease who show high levels of malondialdehyde in their hippocampus region, suggesting a positive correlation between high levels of NO and MDA in injured tissues (Huang et al., 2011 ▶).

The second part of the current study was devoted to show the protective effect of SMN and CLX individually and in combination on the MMF-induced duodenal disorders including histopathological damages and biochemical changes. In the previous study, we showed that the MMF-induced damages in the duodenal region are similar to those of aspirin-created injuries and a local inflammation alongside with oxidative stress were the dominant feature of occurred injuries (Malekinejad et al., 2011 ▶). To this end, in the current study we hypothesized that co-administration of MMF and SMN, MMF and CLX and/or MMF, and a combination of these two compounds may reduce the MMF-induced GI disorders. Undoubtedly, our results showed that both compounds were able to attenuate the MMF-induced detrimental changes in the duodenal region. Although SMN exerted its protective effect at all three tested doses, its appreciable effect was obtained at 50 mg/kg dose level both in histopathological examinations and in biochemical analyses. There are many studies indicating the antioxidant and anti-inflammatory properties of SMN as we showed in this study (Kiruthiga et al., 2007 ▶; Turgut et al., 2008 ▶). To explain why SMN at 50 mg/kg dose level exerted the best effect, it must be taken into account that flavonoids which possess multiple hydroxyl groups may exert pro-oxidant effect at higher doses too. It is interesting to note that different components of SMN mixture possess more than four hydroxyl groups, which may explain the pro-oxidant effect of SMN at higher dose levels (Abou-Samra et al., 2011 ▶). 

CLX also lowered the MMF-induced inflammatory reactions particularly the neutrophil infiltration and consequently the NO production and MPO activity, confirming the nature of the MMF-induced pathological impact as an inflammation (Parfitt et al., 2006 ▶; Telkes et al., 2011 ▶). 

Another interesting finding of this study was that we demonstrated a synergistic effect between SMN and CLX, when they were co-administered. Although statistically, in all performed experiments, we could not prove this. However, in the biochemical analyses which were in the same direction including the neutrophil infiltration, MPO activity, and MDA production, the synergism between two compounds was confirmed. The histopathological findings did not show any synergistic effect between two given compounds. Additionally, in the animals that received both compounds, the MMF-induced damages including the atrophy of villus were not completely diminished. 

The reason for this discrepancy could be a possible interaction between two tested chemicals and short study period for histopathological damages to be cured. Undoubtedly, to clarify the effectiveness of such a combination therapy, further pharmacokinetics studies during longer experimental period are warranted. 

We showed that in the MMF-induced duodenal disorders, in addition to histopathological damages which are characterized by the villus atrophy and mono- and poly-nuclear cell infiltration, the biochemical alterations including a significant elevation of MPO activity, NO and MDA content along with a remarkable reduction of TTM level in the duodenal region were involved. Our findings indicate that the MMF-induced duodenal damages were reduced by SMN alone or in combination with CLX. This protective effect of SMN may be due to its anti-inflammatory and antioxidant properties. 
